# Supplementary material for: Assessing Urinary Para-Hydroxyphenylacetic Acid as a Biomarker Candidate in Neuroendocrine Neoplasms
Source: Int J Mol Sci. 2024 Nov 16;25(22):12317. doi: 10.3390/ijms252212317 (PMC11594794; doi:10.3390/ijms252212317)

Figure S2. T1/T0 serum Chromogranin A and T1/T0 urinary para-HydroxyPhenylAcetic Acid survival analysis

A

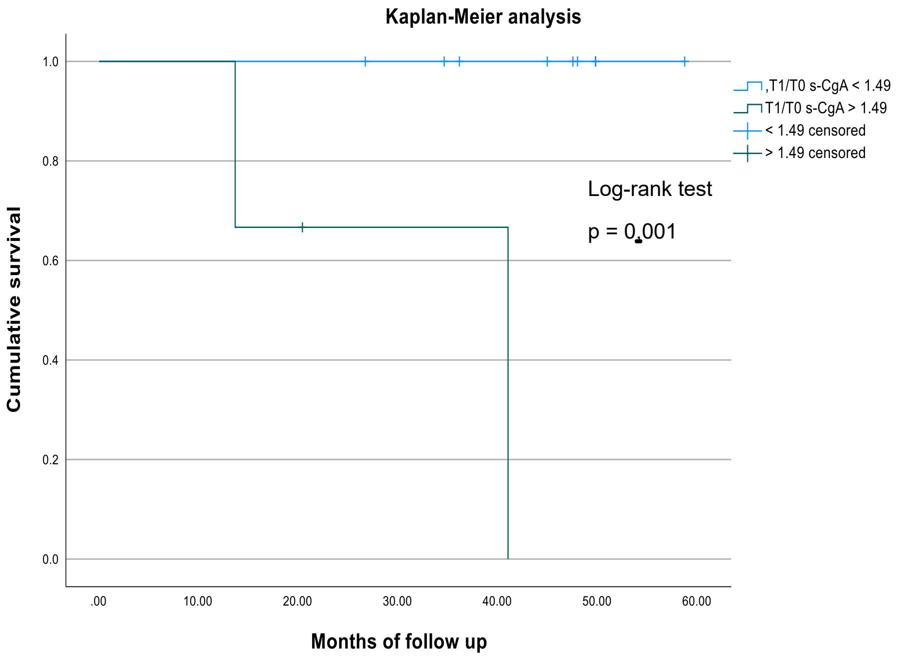

B

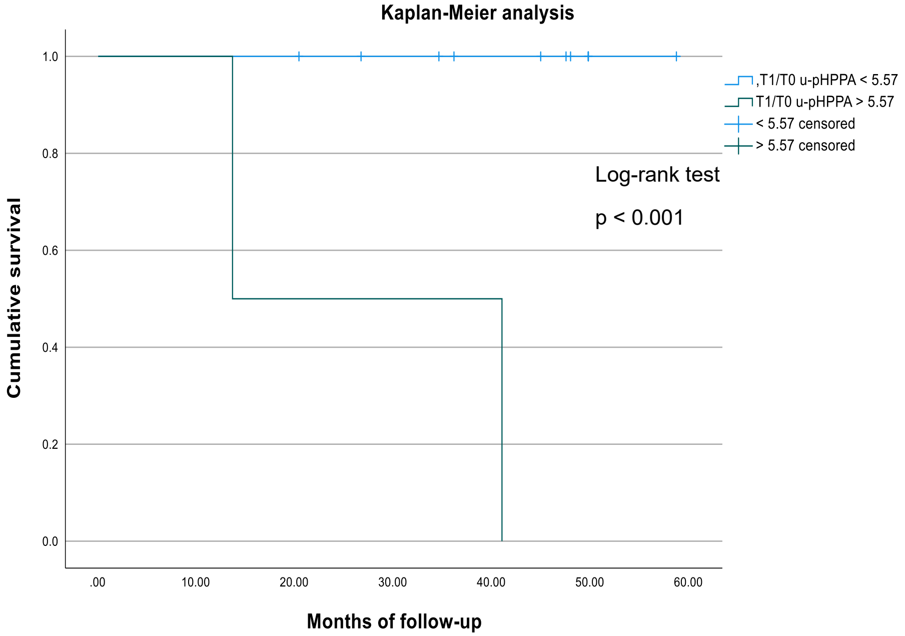

Supplement: Supplementary file 1 [file ijms-25-12317-s001.zip › Supplementary Figure S2.pdf]
