# Supplementary material for: Assessing Urinary Para-Hydroxyphenylacetic Acid as a Biomarker Candidate in Neuroendocrine Neoplasms
Source: Int J Mol Sci. 2024 Nov 16;25(22):12317. doi: 10.3390/ijms252212317 (PMC11594794; doi:10.3390/ijms252212317)
Supplement: Supplementary file 1 [file ijms-25-12317-s001.zip › Supplementary Table S1.pdf]

Supplementary Table S1. Patients characteristics and metastasis/death outcomes							
**Patient**	**Gender**	**Localization**	**Age**	**BMI**	**Metastasis**	**Death outcome**	**Treatment (Somatostatin analogues)**
1	Female	Extrasurrenalic Paraganglioma	24	24.22	No	No	No
2	Male	Gastrointestinal	65	23.39	No	No	No
3	Female	Gastrointestinal	61	26.80	Yes	No	Yes
4	Female	Gastrointestinal	56	28.30	No	No	Yes
5	Female	Gastrointestinal	19	19.47	No	No	No
6	Male	Gastrointestinal	78	18.62	Yes	Yes	Yes
7	Female	Breast	46	18.75	Yes	No	Yes
8	Female	Gastrointestinal	37	29.39	Yes	No	Yes
9	Female	Gastrointestinal	38	21.23	Yes	No	Yes
10	Female	Ovary	60	26.99	Yes	No	Yes
11	Female	Lung	65	22.49	No	No	No
12	Female	Gastrointestinal	46	21.26	No	No	No
13	Female	Gastrointestinal	50	20.60	Yes	No	Yes
14	Male	Lung	55	31.36	Yes	Yes	Yes
