# Supplementary material for: Assessing Urinary Para-Hydroxyphenylacetic Acid as a Biomarker Candidate in Neuroendocrine Neoplasms
Source: Int J Mol Sci. 2024 Nov 16;25(22):12317. doi: 10.3390/ijms252212317 (PMC11594794; doi:10.3390/ijms252212317)
Supplement: Supplementary file 1 [file ijms-25-12317-s001.zip › Supplementary Table S2.pdf]

Supplementary Table S2. T0 and T1 serum and T1/T0 tyrosine levels			
**Patient**	**T_0**	**T_1**	**Normalized T_1/T_0**
1	-0.97	0.51	6.992
2	0.12	0.72	1.452
3	-0.19	-1.03	0.181
4	0.31	0.82	1.329
5	-1.19	-0.016	48.040
6	3.17	0.41	0.372
7	-0.28	-1.22	0.000
8	-1.17	-0.63	13.905
9	0.034	0.36	1.261
10	-1.09	0.31	12.398
11	-1.05	0.014	7.506
12	-0.40	-0.89	0.392
13	-0.42	-0.54	0.849
14	0.29	0.66	1.247
