# Supplementary figures and images for: Assessing Urinary Para-Hydroxyphenylacetic Acid as a Biomarker Candidate in Neuroendocrine Neoplasms
Source: Int J Mol Sci. 2024 Nov 16;25(22):12317. doi: 10.3390/ijms252212317 (PMC11594794; doi:10.3390/ijms252212317)

Figure S1. T1/T0 urinary 5-hydroxyindoleacetic acid and serum Neuro Specific Enolase ROC curves

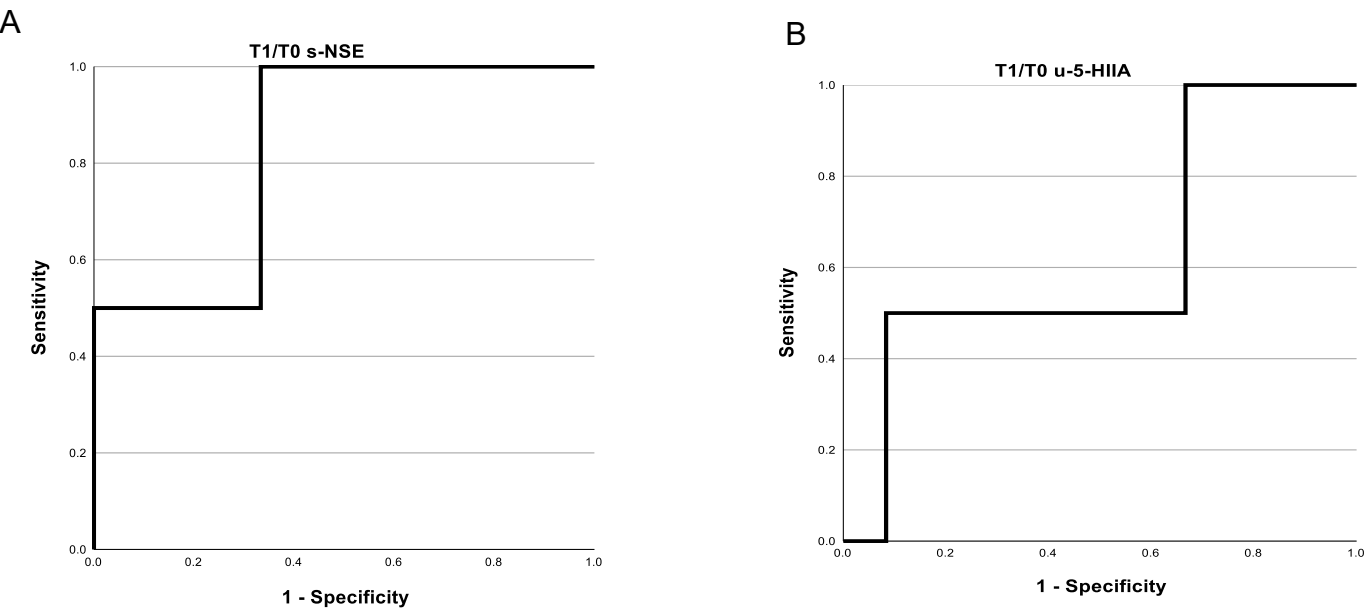

Supplement: Supplementary file 1 [file ijms-25-12317-s001.zip › Supplementary Figure S1.pdf]

Figure S3: Overview of Enriched Metabolite Sets

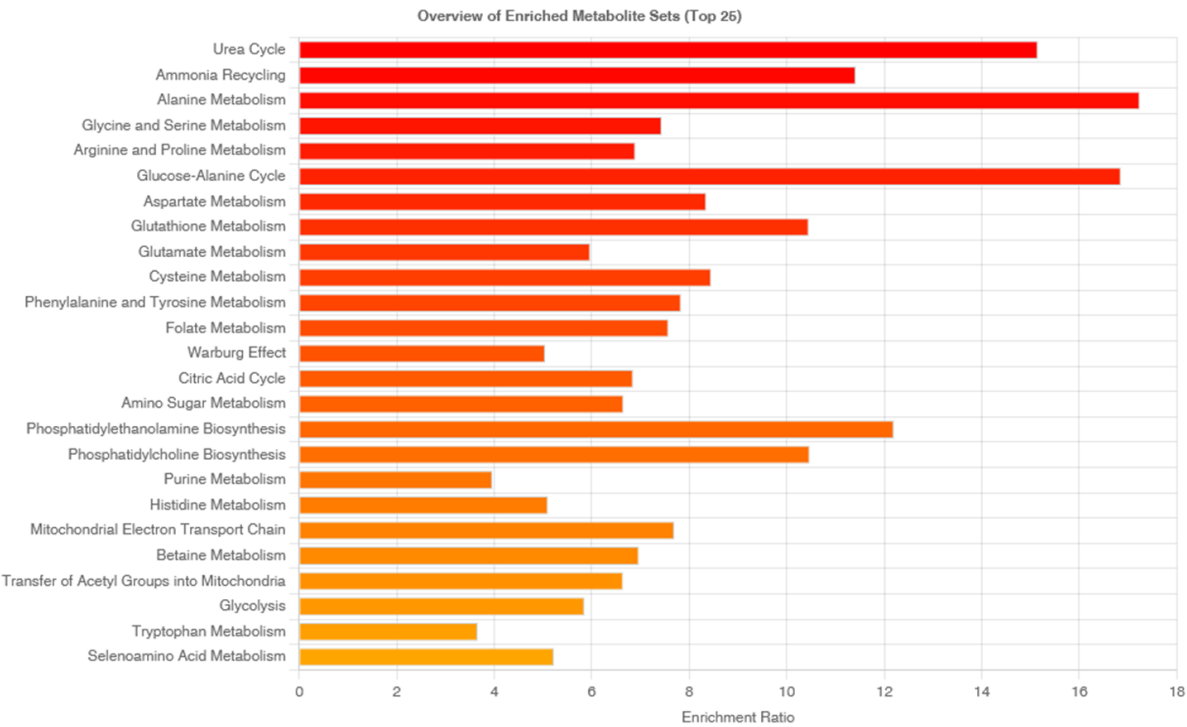

Supplement: Supplementary file 1 [file ijms-25-12317-s001.zip › Supplementary Figure S3.pdf]

Figure S4. T0 serum tyrosine survival analysis

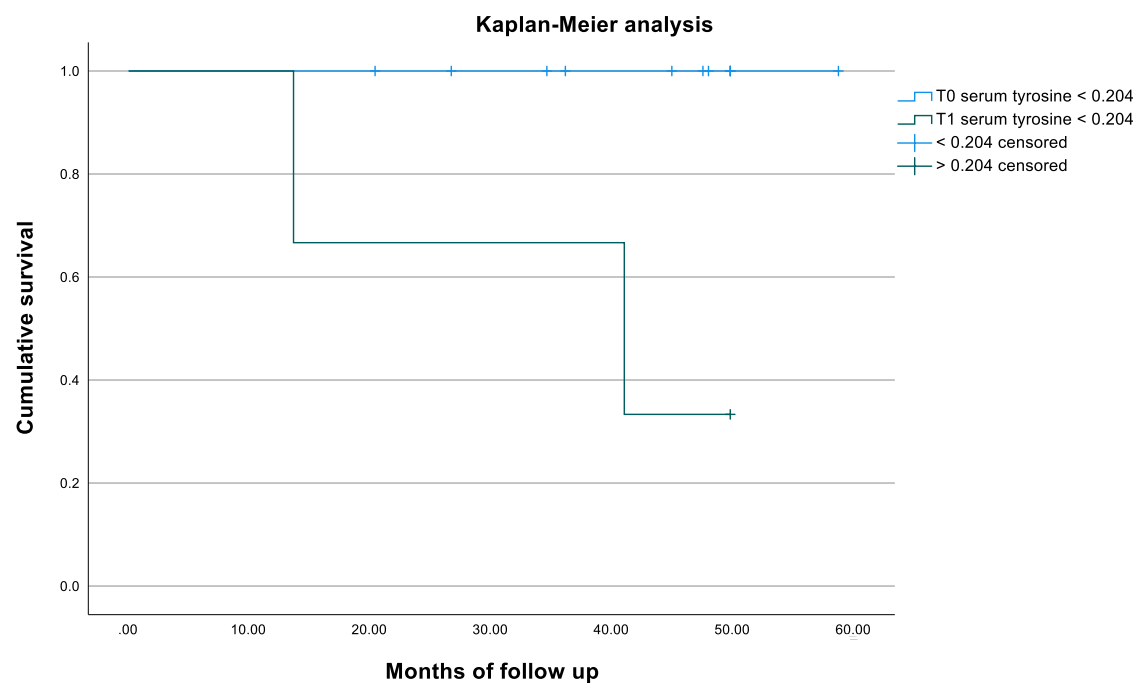

Supplement: Supplementary file 1 [file ijms-25-12317-s001.zip › Supplementary Figure S4.pdf]
